# Supplementary material for: Controlling seizure propagation in large-scale brain networks
Source: PLoS Comput Biol. 2019 Feb 25;15(2):e1006805. doi: 10.1371/journal.pcbi.1006805 (PMC6405161; doi:10.1371/journal.pcbi.1006805)
Supplement: S2 Text — (PDF) [file pcbi.1006805.s002.pdf]

## S2 Text: Topological properties

Topological properties of the structural connectivity matrix of patient **CJ**, examined by means of 6 different graph metrics provided by the general framework of graph theory. These metrics are Efficiency (S2 Fig.), Strength (S2 Fig.), Clustering (S3 Fig.), Degree (S3 Fig.), Betweeness (S4 Fig.), Centrality (S4 Fig.). Neither the epileptogenic zone (highlighted in each panel with a yellow stripe) nor the propagation zone (highlighted in magenta) are characterized by peculiar values of these metrics, thus suggesting that network measures cannot be used to identify biomarkers of these specific brain functions.
