# Supplementary material for: Controlling seizure propagation in large-scale brain networks
Source: PLoS Comput Biol. 2019 Feb 25;15(2):e1006805. doi: 10.1371/journal.pcbi.1006805 (PMC6405161; doi:10.1371/journal.pcbi.1006805)
Supplement: S3 Text — (PDF) [file pcbi.1006805.s003.pdf]

S3 Text: Other patients

Similar results to those shown in the main text has been obtained testing the procedure on the structural connectivity matrices of 15 patients that underwent a presurgical evaluation: this means that for all of them we knew the position of the epileptogenic zone thanks to clinicians' estimation (the clinical characteristics of each patient are given in S1 Table) and we could compare the predicted propagation zone with the one emerging from the analysis of SEEG data coming from implanted electrodes. However the analysis that we have done is not restricted to a particular matrix or epileptogenic zone; on the contrary is has a general applicability, as we have shown in Fig. 6(a) in the main text, and as we are going to show here for other six patients. Even though results depend on the location of the epileptogenic zone and on the subgraph outgoing from this zone, it is always possible to calculate the Linear Stability Analysis of the system, identify the propagation zone and the instability pathways and perform a minimal set of lesions on the connectivity network able to stop the seizure propagation.

Patient FB: FB shows Premotor epilepsy (right side). Engel score II. EZ: Precentral Gyrus. PZ prediction: Postcentral Gyrus, Caudal Middle Frontal Gyrus, Pars opercularis, Superior Frontal Gyrus, Thalamus, Putamen, Paracentral Cortex, Supramarginal Gyrus. PZ clinical prediction: Postcentral Gyrus, Superior Parietal Cortex. Lesions: it is necessary to cut all the links between the Precentral Gyrus and the nodes in the PZ (8) to stop the propagations (see S5 Fig.).

Patient ET: Parietal epilepsy in the left side. Engel score I. EZ regions: Posterior Cingulate Gyrus, Precuneus Cortex. PZ prediction: Isthmus-cingulate cortex, Postcentral Gyrus, Superior Parietal Cortex, Cuneus, Posterior Cingulate Gyrus, Parahippocampal Gyrus. PZ clinical prediction: Postcentral Gyrus, Superior Parietal Cortex. In this case both nodes of the EZ are contributing to destabilize the system, therefore it is necessary to remove the links between each epileptogenic zone and the propagation zones (11 links). In addition to this we have to look also at the propagation flow of the seizure and at the nodes that are immediately recruited due to the strength of their links, but that are not in the PZ (other 2 lesions) (see S6 Fig.).

Patient AC: Temporo-frontal epilepsy in the right side. Engel score III. EZ regions: Lateral Orbito Frontal Cortex, Temporal pole. PZ prediction: Rostral Middle Frontal Gyrus,

Medial Orbito Frontal Cortex, Pars Orbitalis, Insula, Putamen, Pars Triangularis. PZ clinical prediction: Superior Frontal Gyrus, Rostral Middle Frontal Gyrus, (left) Lateral Orbito Frontal Cortex. In this case only one node of the EZ is contributing to the destabilization of the system and it is sufficient to cut the link between LOFC and RMFG to stop the propagation (see S7 Fig.).

Patient GC: Temporal epilepsy in the right side. Engel score III. EZ regions: Amygdala, Hippocampus. PZ prediction: Entorhinal Cortex, Parahippocampal Gyrus, Thalamus, Pallidum, Fusiform Gyrus. PZ clinical prediction: Superior Temporal Gyrus, Temporal Pole, Inferior Temporal Gyrus, Medial Orbito Frontal Cortex, Middle Temporal Gyrus, Parahippocampal Gyrus, Pars orbitalis, Pars triangularis, Rostral Middle Frontal Gyrus, Insula. Hippocampus plays a dominant role with respect to the Amygdala in the recruitment and propagation process, therefore it is sufficient to cut the links between this node and the nodes belonging to the PZ (5 links in total), in addition to the links between Amigdala and Hippocampus, Amigdala and Temporal Pole to stop the seizure. The former link (Amg-TmP) is very strong and the Temporal Pole turns out to be the biggest element of the maximal eigenvector after the nodes belonging to the PZ (see S8 Fig.).

Patient CV: CV shows Supplementary Motor Areas epilesy (left side). Engel score I. EZ regions: Posterior Cingulate Gyrus, Caudal Middle Frontal Gyrus, Superior Frontal Gyrus. PZ prediction: Precentral Gyrus, Caudal Middle Frontal Gyrus, Rostral Middle Frontal Gyrus, (right) Superior Frontal Gyrus, Caudal Anterior Cingulate Cortex, Paracentral Cortex. PZ clinical prediction: Precentral Gyrus, Postcentral Gyrus. It turns out that 1 out of 3 areas in the EZ has a higher impact on the system instability, thus being determinant to the recruitment process. When more that one area constitutes the EZ it is necessary to take into account also the links among the areas, which sustain and enhance the propagation, as testified in this case by the fact that it is necessary to disconnect the link PCG-CMFG to achieve the desired goal. In total, it is sufficient to perform 3 lesions in order to stop the seizure propagation (see S9 Fig.).

Patient RB: Temporal epilepsy in the left side. Engel score III. EZ regions: Amygdala, Hippocampus, Entorhinal cortex, Fusiform gyrus, Temporal Pole, right Entorhinal cortex. PZ prediction: Inferior Temporal Gyrus, Lateral Occipital Cortex, Lingual gyrus, Parahippocampal Gyrus, Cerebellum Cortex. PZ clinical prediction: Superior Temporal Gyrus, Middle Temporal Gyrus, Inferior Temporal Gyrus, Insula, Parahippocampal Gyrus. Links

between all the regions in the EZ and the regions in the PZ must be cut in order to stop the seizure (see S10 Fig.).
